# Inheritance of Monogenic Hereditary Skin Disease and Related Canine Breeds

**DOI:** 10.3390/vetsci9080433

**Published:** 2022-08-15

**Authors:** Pablo Jesús Marín-García, Lola Llobat

**Affiliations:** Department of Animal Production and Health, Veterinary Public Health and Food Sciences and Technology (PASAPTA), Facultad de Veterinaria, Universidad Cardenal Herrera-CEU, CEU Universities, 46113 Valencia, Spain

**Keywords:** skin disorders, genodermatosis, monogenic hereditary dermatosis, ichthyosis, epidermolysis, parakeratosis, mucinosis

## Abstract

**Simple Summary:**

The high prevalence of genetic diseases in dog breeds and the structure of their populations has led to detailed studies of the canine genome, which are important for understanding the origin of these pathologies. The location of certain genes involved in a few autosomal recessive monogenic diseases, including genodermatosis. The most prevalent canine genodermatosis are non-epidermolytic ichthyosis, epidermolytic ichthyosis, and junctional epidermolysis bullosa. Other genodermatoses are nasal paraqueratosis, cutaneous mucinosis, dermoid sinus, lethal acrodermatitis, palmoplantar hyperkeratosis, or exfoliative cutaneous lupus erythematosus. Most of this genodermatosis is associated with a specific and known number of mutations, which have a higher prevalence in certain canine breeds. The main objective of this review is to analyze each of these genodermatoses, the genes and mutations associated with them, and the breeds with the greatest predisposition to suffer from them.

**Abstract:**

The plasticity of the genome is an evolutionary factor in all animal species, including canines, but it can also be the origin of diseases caused by hereditary genetic mutation. Genetic changes, or mutations, that give rise to a pathology in most cases result from recessive alleles that are normally found with minority allelic frequency. The use of genetic improvement increases the consanguinity within canine breeds and, on many occasions, also increases the frequency of these recessive alleles, increasing the prevalence of these pathologies. This prevalence has been known for a long time, but mutations differ according to the canine breed. These genetic diseases, including skin diseases, or genodermatosis, which is narrowly defined as monogenic hereditary dermatosis. In this review, we focus on genodermatosis *sensu estricto*, i.e., monogenic, and hereditary dermatosis, in addition to the clinical features, diagnosis, pathogeny, and treatment. Specifically, this review analyzes epidermolytic and non-epidermolytic ichthyosis, junctional epidermolysis bullosa, nasal parakeratosis, mucinosis, dermoid sinus, among others, in canine breeds, such as Golden Retriever, German Pointer, Australian Shepherd, American Bulldog, Great Dane, Jack Russell Terrier, Labrador Retriever, Shar-Pei, and Rhodesian Ridgeback.

## 1. Introduction

During recent centuries, genetic pathologies in canine breeds have increased considerably, possibly because of a reduction in the effective number of individuals in canine populations due to genetic selection. Such a focus on morphological characteristics has limited the number of alleles, thereby increased consanguinity, and reduced genetic diversity. This has mainly occurred due to inadequate crossing practices, together with insufficient selective pressure on canine well-being and health characteristics [1]. In fact, the effective number of some canine breeds has been estimated at 30–70%, and inbred dogs after two generations have ranged from 1 to 8%, depending on mating practices [2]. Genetic selection has focused on aesthetics rather than function or health, so a small number of breeders have been crossed with closed relatives, producing significantly reduced genetic diversity and increasing the prevalence of specific deleterious alleles [3,4,5]. Dermatological pathologies are no exception, and some have increased considerably in certain breeds. For these, it is important to distinguish genodermatosis (dermatoses of a monogenic origin) from polygenic dermatoses with racial predisposition, the latter being more frequent than others [6]. In this review, we focused strictly on genodermatosis, i.e., monogenic, and hereditary dermatosis.

The high prevalence of genetic diseases in dog breeds and the structure of their populations has led to detailed studies of the canine genome, which are important for understanding the origin of these pathologies. For this reason, it was possible to determine that the canine genome has about 2.42 gigabases (Gb) composed of 20,000 genes distributed on 78 chromosomes, 38 pairs of acrocentric autosomes, and a pair of sex chromosomes: the X chromosome, the largest karyotype with 128 megabases (Mb), and the Y chromosome with the smallest karyotype of 27 Mb [7]. Whole canine genome mapping, sequencing, and linkage analyses has made possible the highlighting of several disease-related sources. First, the high incidence of SINE (short interspersed nuclear element), which are repeated sequences associated with many diseases [8]; the frequent occurrence of SNPs (single nucleotide polymorphisms), which are variations of a single nucleotide in the genome [9], and the location of certain genes involved in several autosomal recessive monogenic diseases, including genodermatosis, have made it possible to reveal multiple causes of disease occurrence.

The canine genodermatosis are mainly non-epidermolytic ichthyosis in the Golden Retriever and the Jack Russell Terrier, epidermolytic ichthyosis in the Norfolk Terrier, junctional epidermolysis bullosa in the German Shorthaired Pointer, and Shar-Pei mucinosis, although other types of canine genodermatosis exist (Table 1).

**Figure 1:**
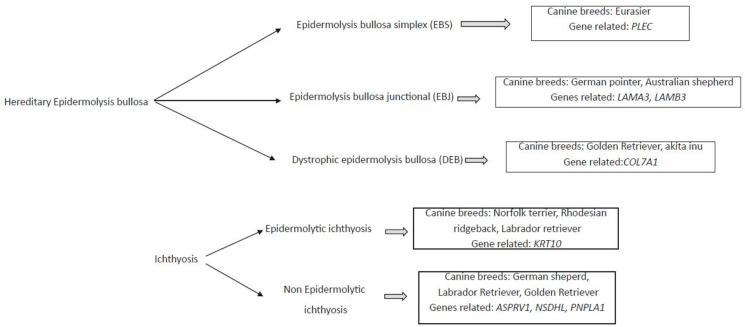
Diagram of mainly monogenic hereditary skin disease, the associated canine breed, and the related genes.

Different genodermatosis has been correlated to canine breed, and the several genes seems to be the responsible for these diseases (Figure 1).

## 2. Hereditary Epidermolysis Bullosa

Hereditary epidermolysis bullosa constitutes a heterogeneous group of hereditary blistering diseases of the skin and mucous membranes [22,23]. These pathologies are characterized by the spontaneous development of vesicles, erosions, and ulcers because of minimal trauma to the excessively fragile dermal–epidermal junction (DEJ) [24]. This group of dermal diseases is classified according to the level of cleavage as epidermolysis bullosa simplex (EBS), epidermolysis bullosa junctional (EBJ), and dystrophic epidermolysis bullosa (DEB). Different dog breeds have been associated with each of these diseases and have presented different types of ulcers. Some genes with recessive autosomal inheritance have been associated with them (Table 2).

Epidermolysis bullosa simplex (EBS) is a skin disease, in which the keratinocytes or basal and suprabasal are related [25]. This disease is not unique to dogs, so different subtypes of EBS have also been observed in humans, and different genes play a role [28,29,30,31,32,33,34,35,36]. However, in dogs, only two genes have been associated with it. The mutation of the *PLEC* gene has been associated so far with EBS in Eurasier dogs [25]. The product of the *PLEC* gene is plectin, a 500 kDa protein found in skin and other tissues, such as bone, muscle, and the nervous system [37]. There are likely different isoforms of plectin that are cell-type dependent and/or developmentally regulated [38]. Mauldin et al. (2017) demonstrated that in dogs with a homozygous G-to-A variant in the *PLEC* gene, a tryptophan is converted to a premature stop codon in exon 27, resulting in this disease with autosomal recessive inheritance [39]. On the other hand, Olivry et al. (2012) showed the association between a single mutation in the first intron of *PKP1* gene. This single mutation results in a premature stop codon, and the absence of the protein plakophilin-1, a protein that stabilizes desmosomes in the skin [40,41]. The inheritance of this mutation was also autosomal recessive, and this detection occurred in dog breed Chesapeake Bay Retriever, resulting in an ectodermal dysplasia-skin fragility syndrome [40].

In epidermolysis bullosa junctional (EBJ), cleft formation occurs through the lamina lucida of the basement membrane zone. Affected individuals exhibit blisters, deep erosions, and ulcers [22]. In humans, mutations in several genes have been associated with this pathology, including genes encoding subunits of integrins (*ITGA6*, *ITGB4*, and *ITGA3*), collagen (*COL17A1*), and laminin 332 (*LAMA3*, *LAMB3*, and *LAMC2*) [42,43]. Recently, mutations in the *LAMA3* and *LAMB3* genes have been associated with EBJ in Australian shepherd dogs [11,26]. In the study by Kiener et al. (2020), a *LAMB3*:c.1174T > C mutation was reported as the cause of EBJ, suggesting an autosomal recessive inheritance of this mutation [11]. The *LAMB3* gene encodes the β3-polypeptide chain of laminin-1 [44] and has been associated with the progression of several human tumors [45]. The recent study by Herrmann et al. (2021) reported a *LAMA3* mutation associated with EBJ and severe upper respiratory disease in Australian Shepherd dogs [26]. This mutation (Asp2867Val) results in a missense variant in the laminin-α3 chain with autosomal recessive inheritance. Other mutations in the same gene have been found in the German Pointer dog breed associated with EBJ. Specifically, an insertion of repetitive satellite DNA in intron 35 of this gene has been associated with EBJ [10,27]. This insertion results in an α3-pre-messenger RNA that is not well matured and a decrease in laminin 5 expression, thereby impairing adhesion and the clonogenic potential of the EBJ keratinocytes.

Finally, in dystrophic epidermolysis bullosa (DEB), blistering occurs in the sublamina densa, and the skin and mucosa are extremely sensitive. The blisters heal with scarring, and end with progressive disability and the deformation of the fingers [46]. This disease, which affects dogs, sheep, cattle, cats, and humans, is caused by mutations in the *COL7A1* gene, which encodes collagen type VII [47]. A total of 500 mutations of this gene have been associated with DEB, and the severity of the phenotype depends on the type of mutations and their location [48]. Most of these mutations were observed in the golden retriever, although Nagata et al. (1995) reported a case of DEB in Akita Inu dog breed [12]. The authors did not perform a genetic study on the animal, and the results they observed when analyzing the bladders by electronic microscopy and immunohistochemistry were comparable to those in humans and other dogs suffering with this disease [12]. Several studies reported new therapies to control and eradicate this disease. In one study, canine keratinocytes were used to generate autologous epidermal layers in dogs with homozygous missense mutation in the *COL7A1* gene, which expressed an aberrant protein, with good results [49]. Other authors attempted gene therapy with retroviral vectors [50]. Recently, Gretzmeier et al. (2021) published good results when recombinant protein collagen VII (C7) was administered to mice and dogs [51].

## 3. Ichthyosis

The term ichthyosis describes rare congenital or hereditary pathologies caused by primary defects in the formation of the stratum corneum [52]. This ichthyosis could be epidermolytic or non-epidermolytic, depending on whether they are vacuoles and lysis of keratinocytes within the spinous and granular cell layers [53]. Epidermolytic ichthyosis has been described in the Norfolk Terrier concerning a mutation to the epidermal keratin gene (*KRT10*) with autosomal recessive inheritance [54], although the same pathology has been described in the Rhodesian Ridgeback and Labrador Retriever.

However, the most common ichthyosis is non-epidermolytic and presents autosomal recessive inheritance. In humans, there are X-linked dominant forms [55], but in dogs these forms are yet to be documented [52], with two exceptions. The first is the autosomal dominant inheritance of mutation c.1052C > T in the *ASPRV1* gene in the German Shepherd [56]; the second is the deletion identified in the *NSDHL* gene of two female Labrador Retrievers, which encoded an NAD(P)-dependent steroid dehydrogenase-like protein related to cholesterol biosynthesis and with monogenic X-chromosomal semidominant inheritance [57]. Different mutations in several genes have been related (Table 3).

One of the canine breeds most affected by non-epidermolytic ichthyosis is the golden retriever. In this breed, the clinical signs include a generalized scaling and hyperpigmented and rough ventral glabrous skin. The histopathology shows a laminated orthokeratosis and an epidermal hyperkeratosis without significant involvement of the stratum granulosum [39,60]. The *PNPLA1* variant that produces this pathology reached more than 50% frequency in the breeding population now of identification [17]. The frequencies of genotypes are estimated around 32% in affected dogs (homozygous recessives), 49% heterozygous, and 20% homozygous dominant, thus clean of defective variants [61]. More recently, these frequencies have been estimated at 21% in wild-type, 48% in heterozygous, and 31% in recessive homozygous [18]. The PNPLA protein family has nine patatin-like phospholipases (PNPLA1-PNPLA9) with lipolytic and acyltransferase activities and are related to lipid metabolism [62,63]. In humans, five mutations of *PNPLA1* caused autosomal recessive congenital ichthyosis, which affects the composition and organization of epidermal lipids. All five mutations provoke a *PNPLA1* amino acid change [64]. In dogs, specifically Golden Retrievers with this mutation, an indel in exon 8 is reported to cause non-epidermolytic ichthyosis by GWAS analysis [17]. To evaluate the efficacy of treatment with shampoo and lotion containing gluconolactone and other hydroxylated acids, a prospective study was carried out, and the results were encouraging: the extension and size of the scales was reduced between 60 and 75% after 14 and 30 days of treatment, respectively [65]. Recently, Kiener et al. (2021) reported a *ABHD5* gene frameshift deletion in Golden Retrievers with non-epidermolytic ichthyosis [59]. The mutation is a 14 bp deletion that provokes a frameshift that alters the last 14 codons. The *ABHD5* gene encodes an acyltransferase related to lipid metabolism, and defects in this gene are related to Chanarin–Dorfman syndrome, a neutral lipid storage disease with ichthyosis [66,67]. To date, these mutations have not been reported in other breeds; however, they have presented mutations related to non-epidermolytic ichthyosis. For example, a variant of *ASPRV1* gene has been found in German Shepherds [56]. This gene encodes a retroviral-like protease involved in profilaggrin-to-filaggrin processing and plays a relevant role in skin barrier formation [68]. The missense variant of c.1052T < C has found in this breed, which affects a conserved residue and produces the amino acid change Leu351Pro. This change provokes a deficient ASPRV1 protein, which produces a lower level of stratum corneum hydration [69]. In the American Bulldog, mutations in the *NIPAL4* gene are related to non-epidermolytic ichthyosis [14,70] and in humans to autosomal recessive congenital ichthyosis [71]. In dogs, the frameshift deletion of the *NIPAL4* gene produces a premature stop codon that results in a truncated and defective NIPAL4 protein [59]. This protein seems to have a relevant role in lipid metabolism, and it is associated with keratins and desmosomes in the epidermis [72]. Therefore, animals with deficient NIPAL4 protein fail to form normal lamellar bilayers, leading to the appearance of the typical clinical signs of non-epidermolytic ichthyosis [73].

In Great Danes, a mutant transcript of the *SLC27A4* gene has been correlated to the ichthyosis phenotype by sequence analysis [15]. The mutation provokes an in-frame loss of 54 bp in exon 8, that probably affects protein expression. The mutant dogs presented a truncated protein levels elevated. The SLC27A4 protein has acyl-CoA synthetase activity, which is related to fatty-acid and phospholipid synthesis and, consequently, to lipid metabolism [74] and fatty-acid transport in the cell membrane [75]. Some mutations in the *SLCC27A4* gene have been associated with ichthyosis in human patients [76,77,78], so it is probable that the mutation in Great Danes is not the only one in this gene related to the disease in dogs. In Jack Russell Terriers, [16] related the lamellar ichthyosis to a LINE-1 insertion in the transglutaminase 1 (*TGM1*) gene, which encodes an enzyme with a role in cornified envelope formation, and 30–40% of humans with non-epidermolytic (lamellar) ichthyosis present mutations in this gene [79]. The authors identified a LINE-1 insertion in this gene related to non-epidermolytic ichthyosis phenotype as found in humans.

Finally, mutations in *CERS3* have been related to autosomal recessive congenital ichthyosis in humans [80]. Even though these mutations have not yet been found in dogs, it would be interesting to analyze the prevalence of these mutations to see if the phenotype they produce is like that of humans. This gene encodes a protein with a relevant role in sphingolipid metabolism and is essential for the maintenance of epidermal lipid homeostasis. In fact, mutations found in other human genes related to ichthyosis have been related to different types of canine genodermatosis. For example, Caroppo et al. (2020) recently reported a novel keratin 1 (*KRT1*) c.1433A > G mutation related to human epidermolytic ichthyosis [81]. Other mutations in the same gene [82] or others of the same family [83] have been related to human ichthyosis and to different canine skin pathologies: epidermolytic ichthyosis, epidermolytic hyperkeratosis, and nasal parakeratosis [54,84,85].

## 4. Other Genodermatosis

**Figure 2:**
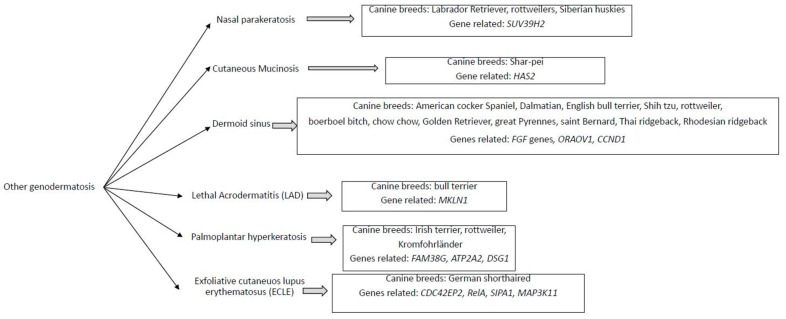
Diagram of other genodermatosis found the associated canine breed and the genes candidates to be responsible.

Other genodermatosis have been described in different canine breeds and the genes candidates have been studied (Figure 2).

Nasal parakeratosis is a variety of genodermatosis characterized by a thick, slightly verrucous, brown scale on the nasal planum with variable depigmentation [86]. Detected in Labrador Retrievers, Rottweilers and Siberian Huskies, this pathology is characterized by the accumulation of serum in the nasal epidermis and numerous intracorneal vacuoles [87,88]. Afterwards, several studies connected a mutation in the *SUV39H2* gene with this pathology in Labrador Retrievers [19] and Greyhounds [39]. The gene encoded histone 3 methyltransferase, which helps regulate protein stability and activity, protein–protein interactions, and epigenetic silencing [89,90]. Jagannathan et al. (2013) detected a missense variant c.972T > G, with the amino acid change Asn324Lys in Labrador Retrievers affected by nasal parakeratosis [19]. Later, the same group related nasal parakeratosis in Greyhounds with a 4 bp deletion at the 5′-splice site of intron 4 [39]. These data suggest that mutations in the *SUV39H2* gene could be related to nasal parakeratosis in different breeds. More recently, Bannoehr et al. (2020) analyzed Labrador Retrievers affected by nasal parakeratosis and the c.972T > G mutation in the *SUV39H2* gene [85]. The results showed an up-regulation of genes that encode keratins 1, 10, and 14, although their expression did not cause changes in the nasal planum, suggesting that the SUV39H2 enzyme affected several genes or pathways related to epidermal differentiation.

Cutaneous mucinosis was described for the first time in seven Shar-Peis that presented asymptomatic nodules, papules, or plaques on the skin or oral mucosa and an excess accumulation of mucin within the dermis or submucosa [91]. Immunohistochemical techniques revealed the sulphated acid glycosaminoglycans in mast cell granules and other mast cell subtypes [92,93]. An analysis of those with mucinosis revealed a high serum concentration of hyaluronic acid, the main component of mucin [94]. In fact, there was a higher transcription of hyaluronan synthase 2 and protein expression in fibroblasts [95,96], indicating a relationship between cutaneous mucinosis and the genetic cause related to this enzyme. In humans, the *HAS2* gene expresses a protein that correlates with malignant transformation [97]. Its activity is regulated by the phosphorylation of protein kinase C [98] and adenosine monophosphate-activated protein kinase [99], which can induce *HAS2* transcript accumulation in dermal fibroblasts [100]. HAS proteins facilitated the extrusion of hyaluronan to the extracellular space [101], and this could explain the relationship between mucinosis in the Shar-Peis and high levels of HAS2 protein expression and hyaluronan accumulation. However, more study is necessary to determine the causal mutation related to this genodermatosis.

Dermoid sinus is caused by incomplete separation of the skin and neural tube during embryonic development [102]. This congenital malformation has been found in different species, including humans [103,104] and dogs. Up to now, the canine breeds where it has been reported are the American Cocker Spaniel [105], Dalmatian [106], English Bull Terrier [107], Shih Tzu [108], Rottweiler [109], Boerboel Bitch [110], Chow Chow [111], Golden Retriever [112], Great Pyrenees [113], Saint Bernard [114], Thai Ridgeback [21] and Rhodesian Ridgeback [115,116,117,118]. In the last one, several authors concluded that the Ridgeback has an autosomal dominant mutation related to dermoid sinus emergence [21,119]. This mutation is a 133 Kb duplication of three fibroblast growth factor (FGF) genes (*FGF3*, *FGF4*, *FGF19*), the oral cancer overexpressed gene (*ORAOV1*), and the *CCND1* gene, which encodes cyclin D1 [21]. The FGF family comprises 17 members with mitogenic or metabolic activity (FGF19, FGF21 and FGF23). The FGFs with mitogenic activity play a critical role in metabolic development, while those with metabolic activity play a role in its regulation [120]. On the other hand, the *ORAOV1* gene is associated with different types of cancer in human patients [121,122,123,124] because it is a regulator of the cell cycle and apoptosis [125]. Furthermore, the expression of cyclin D1 (encoded by *CCND1* gene) is reduced in ORAOV1-silenced cells [126], which could indicate dysregulation of the cell cycle mediated by this gene and cyclin D1 in animals with this mutation. However, few studies have been carried out in this regard.

Lethal acrodermatitis (LAD) is a genetically determined metabolic disease of Bull Terriers that was found in the U.S. in the 1980s [125]. This disease is not exclusively a pathology of the skin, so different characteristics are also reported: stunting, splayed digits, eating difficulties, and increased susceptibility to microbial infections [125,127]. After analyzing the liver-soluble proteome, 13 differentially expressed proteins, including chaperones, for calcium binding, energy metabolism, and inflammatory response were identified [128]. In a genome-wide association study and haplotype analysis, Bauer et al. (2018) showed a splice-region variant in the *MKLN1* gene associated with the presence of disease [129].

Palmoplantar hyperkeratosis in Irish Terriers was associated with autosomal recessive inheritance in a retrospective analysis by Binder et al. (2000) and it was associated with a complex mutation in the *KRT16* gene, corresponding to an insertion/deletion of four nucleotides downstream in exon 6 [130]. The last one is a good model for human focal non-epidermolytic palmoplantar keratoderma (FNEPPK) [131]. This disease is characterized by the abnormal development of the footpad epidermis, and the affected dogs developed smooth parchment-like footpads at the age of six months. The pad epidermis hardened and grew lateral cone-like protrusions of up to 5 mm in diameter and developed fissures and cracks, which predisposed the affected dogs to secondary infections [132]. Several mutations in different genes have been associated with this disease, including mutations in the genes encoding keratin 2 and 9, and desmoglein 1 [132], and a variant is the missense c.155G > C in the *FAM83G* gene, which encodes a protein that has a largely unknown function [133]. In this same breed, the heterozygous SINE insertion into the *ATP2A2* gene is associated with Darier canine disease, a rare form of genodermatosis that affects different breeds [134,135]. Concretely, Linek et al. (2020) showed a demarcated ulcerative and crusting lesion in the ear canal in one Irish Terrier, related to canine Darier disease [135]. The dog presented a splicing defect and marker allelic imbalance in *ATP2A2* mRNA from skin. In the Kromfhrländer canine breed, a variant FAM83G:c155G > C has been related to palmoplantar hyperkeratosis [136], and Backel et al. (2020) found recently a *DSG1* gene variant in a single male rottweiler [137]. This gene encoding desmoglein 1 and variants of this gene have been related to palmoplantar keratoderma in humans [138]. Therefore, future studies about the relationship between this gene and this disease in different dog breeds would be interesting.

Exfoliative cutaneous lupus erythematosus (ECLE) has been described in German Shorthaired Pointer dogs with monogenic autosomal recessive inheritance [139]. The treatment with ciclosporin, hydroxychloroquine, and adalimumab does not seem to have good long-term results [140], whereas the treatment with mycophenolate mofetil seems to achieve a complete remission of the disease [141]. This disease seemed to be related to a SNP allele on canine chromosome 18 [139]. These authors concluded that different candidate genes could be related to ECLE, including genes *CDC42EP2* (a Rho GPase regulates downstream effector proteins for the assembly of the actin cytoskeleton [142]), *RelA* (part of the KFkB complex, involved in immune processes [143]), *SIPA1* (involved cell cycle progression [144]), and *MAP3K11*, which is required for the activation of JNK, p38, and ERK [145]. Leeb et al. (2020) realized a genome-wide association study and they concluded that the p. Pro480Thr mutation in the *UNC93B1* gene is causing ECLE in dogs [146].

Finally, hereditary sensory and autonomic neuropathies (HSAN) should be noted in this review, as lesions (gross or microscopical) are only detected in the skin. These diseases are characterized by progressive sensory loss, chronic skin ulcerations, and nail dystrophic changes [147]. Several mutations have been correlated with these HSAN in canine breeds. In Border Collies, the inversion disrupting *FAM134B* and the missense variant in the *RETREG1* (reticulophagy regulator 1) gene and are associated with HSAN has been detected in Border Collies, Spaniels, and Pointers [148,149,150]. The last one variant has also been associated with these diseases in other canine breeds, such as Spaniels and Pointers [150]. In Siberian Huskies, the polyneuropathy has been related to five different mutations in *NDRG1*, *ARHGEF10*, and *RAB3GAP1* genes [151], and a point mutation in a lincRNA of *GDNF* gene has been associated with HSAN in French Spaniels by genome-wide association study (GWAS) [152].

## 5. Conclusions

Hereditary diseases affect a great number of canine breeds. These diseases include genodermatosis, narrowly defined as monogenic hereditary dermatoses, and could be epidermolysis, ichthyosis, nasal parakeratosis, mucinosis, or dermoid sinus. All these present with a genetic inheritance in certain canine breeds and the specific canine genodermatosis of a dog is life-threatening and the animal welfare is markedly reduced, which could be treated with reducing the skin problems. In some, the causal mutation and its type of inheritance is well known for certain breeds, while for others, only the breed with the highest prevalence of the pathology is known. Several studies are necessary to elucidate the causal mutations and their prevalence in different breeds to incorporate the studies of genetic selection programs of the different breeds to minimize or eradicate this type of dermatologic disease for which there is still no definitive cure.

## Figures and Tables

**Table 1:** Genodermatosis with known causative genetic variants in dog breeds.

Phenotype	Breed	Inheritance ^1^	Reference
Junctional epidermolysis bullosa	German Pointer, Australian Shepherd	AR	[10,11]
Epidermolysis bullosa dystrophic	Golden Retriever, Akita Inu	AR	[12,13]
Ichthyosis	American Bulldog, Great Dane, Jack Russell Terrier, Golden Retriever	AR	[14,15,16,17,18]
Nasal parakeratosis	Labrador Retriever	AR	[19]
Mucinosis	Shar-Pei	ASD	[20]
Dermoid sinus	Rhodesian Ridgeback	AR	[21]

^1^ AR: autosomal recessive inheritance; ASD: autosomal semi-dominant inheritance.

**Table 2:** Classification of canine epidermolysis, related ulcerations, and the associated genes and canine breeds.

Classification Epidermolysis	Dog Breeds	Type of Ulceration	Gene Associated	Reference
Epidermolysis bullosa simplex (EBS)	Eurasier dog	Multifocal ulcers	*PLEC*	[25]
Epidermolysis bullosa junctional (EBJ)	German Pointer, Australian Shepherd	Skin and mucous membrane ulcers	*LAMA3*, *LAMB3*	[10,11,26,27]
Dystrophic epidermolysis bullosa (DEB)	Golden Retriever, Akita Inu	Oral cavity ulcers	*COL7A1*	[12,13]

**Table 3:** Ichthyosis with known gene associated in canine breed.

Dog Breeds	Gene Associated	Inheritance ^1^	Reference
Golden Retriever	*ABHD5*	AR	[58]
	*PNPLA1*	AR	[17]
German Shepherd	*ASPRV1*	AD	[56]
American Bulldog	*NIPAL4*	AR	[59]
Great Dane	*SLC27A4*	AR	[15]
Jack Russell Terrier	*TGM1*	AR	[16]

^1^ AR: autosomal recessive inheritance; AD: autosomal dominant inheritance.

## Data Availability

Not applicable.

## References

[1] Wade C.M. (2011). Inbreeding and Genetic Diversity in Dogs: Results from DNA Analysis. Vet. J..

[2] Leroy G., Baumung R. (2011). Mating Practices and the Dissemination of Genetic Disorders in Domestic Animals, Based on the Example of Dog Breeding. Anim. Genet..

[3] Cruz F., Vilà C., Webster M.T. (2008). The Legacy of Domestication: Accumulation of Deleterious Mutations in the Dog Genome. Mol. Biol. Evol..

[4] Mooney J.A., Yohannes A., Lohmueller K.E. (2021). The Impact of Identity by Descent on Fitness and Disease in Dogs. Proc. Natl. Acad. Sci. USA.

[5] Makino T., Rubin C.-J., Carneiro M., Axelsson E., Andersson L., Webster M.T. (2018). Elevated Proportions of Deleterious Genetic Variation in Domestic Animals and Plants. Genome Biol. Evol..

[6] Leeb T., Roosje P., Welle M. (2021). Genetics of Inherited Skin Disorders in Dogs. Vet. J..

[7] Switonski M., Szczerbal I., Nowacka J. (2004). The Dog Genome Map and Its Use in Mammalian Comparative Genomics. J. Appl. Genet..

[8] Wang W., Kirkness E.F. (2005). Short Interspersed Elements (SINEs) Are a Major Source of Canine Genomic Diversity. Genome Res..

[9] Lindblad-Toh K., Wade C.M., Mikkelsen T.S., Karlsson E.K., Jaffe D.B., Kamal M., Clamp M., Chang J.L., Kulbokas E.J., Zody M.C. (2005). Genome Sequence, Comparative Analysis and Haplotype Structure of the Domestic Dog. Nature.

[10] Capt A., Spirito F., Guaguere E., Spadafora A., Ortonne J.-P., Meneguzzi G. (2005). Inherited Junctional Epidermolysis Bullosa in the German Pointer: Establishment of a Large Animal Model. J. Investig. Dermatol..

[11] Kiener S., Laprais A., Mauldin E.A., Jagannathan V., Olivry T., Leeb T. (2020). LAMB3 Missense Variant in Australian Shepherd Dogs with Junctional Epidermolysis Bullosa. Genes.

[12] Nagata M., Shimizu H., Masunaga T., Nishikawa T., Nanko H., Kariya K., Washizu T., Ishida T. (1995). Dystrophic Form of Inherited Epidermolysis Bullosa in a Dog (Akita Inu). Br. J. Dermatol..

[13] Magnol J.-P., Pin D., Palazzi X., Lacour J.-P., Gache Y., Meneguzzi G. (2005). Characterization of a canine model of dystrophic bullous epidermolysis (DBE). Development of a gene therapy protocol. Bull. Acad. Natl. Med..

[14] Mauldin E.A., Wang P., Evans E., Cantner C.A., Ferracone J.D., Credille K.M., Casal M.L. (2015). Autosomal Recessive Congenital Ichthyosis in American Bulldogs Is Associated with NIPAL4 (ICHTHYIN) Deficiency. Vet. Pathol..

[15] Metzger J., Wöhlke A., Mischke R., Hoffmann A., Hewicker-Trautwein M., Küch E.-M., Naim H.Y., Distl O. (2015). A Novel SLC27A4 Splice Acceptor Site Mutation in Great Danes with Ichthyosis. PLoS ONE.

[16] Credille K.M., Minor J.S., Barnhart K.F., Lee E., Cox M.L., Tucker K.A., Diegel K.L., Venta P.J., Hohl D., Huber M. (2009). Transglutaminase 1-Deficient Recessive Lamellar Ichthyosis Associated with a LINE-1 Insertion in Jack Russell Terrier Dogs. Br. J. Dermatol..

[17] Grall A., Guaguère E., Planchais S., Grond S., Bourrat E., Hausser I., Hitte C., Le Gallo M., Derbois C., Kim G.-J. (2012). PNPLA1 Mutations Cause Autosomal Recessive Congenital Ichthyosis in Golden Retriever Dogs and Humans. Nat. Genet..

[18] Graziano L., Vasconi M., Cornegliani L. (2018). Prevalence of PNPLA1 Gene Mutation in 48 Breeding Golden Retriever Dogs. Vet. Sci..

[19] Jagannathan V., Bannoehr J., Plattet P., Hauswirth R., Drögemüller C., Drögemüller M., Wiener D.J., Doherr M., Owczarek-Lipska M., Galichet A. (2013). A Mutation in the SUV39H2 Gene in Labrador Retrievers with Hereditary Nasal Parakeratosis (HNPK) Provides Insights into the Epigenetics of Keratinocyte Differentiation. PLoS Genet..

[20] von Bomhard D., Kraft W. (1998). Idiopathic mucinosis cutis in Chinese Shar pei dogs: Epidemiology, clinical features, histopathologic findings and treatment. Tierarztl. Prax. Ausg. K Kleintiere Heimtiere.

[21] Salmon Hillbertz N.H.C., Isaksson M., Karlsson E.K., Hellmén E., Pielberg G.R., Savolainen P., Wade C.M., von Euler H., Gustafson U., Hedhammar A. (2007). Duplication of FGF3, FGF4, FGF19 and ORAOV1 Causes Hair Ridge and Predisposition to Dermoid Sinus in Ridgeback Dogs. Nat. Genet..

[22] Fine J.-D., Bruckner-Tuderman L., Eady R.A.J., Bauer E.A., Bauer J.W., Has C., Heagerty A., Hintner H., Hovnanian A., Jonkman M.F. (2014). Inherited Epidermolysis Bullosa: Updated Recommendations on Diagnosis and Classification. J. Am. Acad. Dermatol..

[23] Medeiros G.X., Riet-Correa F. (2015). Epidermolysis Bullosa in Animals: A Review. Vet. Dermatol..

[24] Uitto J., Pulkkinen L. (2001). Molecular Genetics of Heritable Blistering Disorders. Arch. Dermatol..

[25] Mauldin E.A., Wang P., Olivry T., Henthorn P.S., Casal M.L. (2017). Epidermolysis Bullosa Simplex in Sibling Eurasier Dogs Is Caused by a PLEC Non-Sense Variant. Vet. Dermatol..

[26] Herrmann I., Linder K.E., Meurs K.M., Friedenberg S.G., Cullen J., Olby N., Bizikova P. (2021). Canine Junctional Epidermolysis Bullosa Due to a Novel Mutation in LAMA3 with Severe Upper Respiratory Involvement. Vet. Dermatol..

[27] Frattini S., Polli M., Cortellari M., Negro A., Bionda A., Riva J., Rizzi R., Marelli S., Crepaldi P. (2021). Genetic Trend of the Junctional Epidermolysis Bullosa in the German Shorthaired Pointer in Italy. Vet. Rec. Open.

[28] Pigors M., Schwieger-Briel A., Leppert J., Kiritsi D., Kohlhase J., Bruckner-Tuderman L., Has C. (2014). Molecular Heterogeneity of Epidermolysis Bullosa Simplex: Contribution of EXPH5 Mutations. J. Investig. Dermatol..

[29] McGrath J.A., Stone K.L., Begum R., Simpson M.A., Dopping-Hepenstal P.J., Liu L., McMillan J.R., South A.P., Pourreyron C., McLean W.H.I. (2012). Germline Mutation in EXPH5 Implicates the Rab27B Effector Protein Slac2-b in Inherited Skin Fragility. Am. J. Hum. Genet..

[30] McGrath J.A., Mellerio J.E. (2010). Ectodermal Dysplasia-Skin Fragility Syndrome. Dermatol. Clin..

[31] Pigors M., Kiritsi D., Cobzaru C., Schwieger-Briel A., Suárez J., Faletra F., Aho H., Mäkelä L., Kern J.S., Bruckner-Tuderman L. (2012). TGM5 Mutations Impact Epidermal Differentiation in Acral Peeling Skin Syndrome. J. Investig. Dermatol..

[32] Pigors M., Kiritsi D., Krümpelmann S., Wagner N., He Y., Podda M., Kohlhase J., Hausser I., Bruckner-Tuderman L., Has C. (2011). Lack of Plakoglobin Leads to Lethal Congenital Epidermolysis Bullosa: A Novel Clinico-Genetic Entity. Hum. Mol. Genet..

[33] Bolling M.C., Veenstra M.J., Jonkman M.F., Diercks G.F.H., Curry C.J., Fisher J., Pas H.H., Bruckner A.L. (2010). Lethal Acantholytic Epidermolysis Bullosa Due to a Novel Homozygous Deletion in DSP: Expanding the Phenotype and Implications for Desmoplakin Function in Skin and Heart. Br. J. Dermatol..

[34] Hobbs R.P., Han S.Y., van der Zwaag P.A., Bolling M.C., Jongbloed J.D.H., Jonkman M.F., Getsios S., Paller A.S., Green K.J. (2010). Insights from a Desmoplakin Mutation Identified in Lethal Acantholytic Epidermolysis Bullosa. J. Investig. Derm..

[35] Jonkman M.F., Pasmooij A.M.G., Pasmans S.G.M.A., van den Berg M.P., Ter Horst H.J., Timmer A., Pas H.H. (2005). Loss of Desmoplakin Tail Causes Lethal Acantholytic Epidermolysis Bullosa. Am. J. Hum. Genet..

[36] Kiritsi D., Cosgarea I., Franzke C.-W., Schumann H., Oji V., Kohlhase J., Bruckner-Tuderman L., Has C. (2010). Acral Peeling Skin Syndrome with TGM5 Gene Mutations May Resemble Epidermolysis Bullosa Simplex in Young Individuals. J. Investig. Dermatol..

[37] Castañón M.J., Walko G., Winter L., Wiche G. (2013). Plectin-Intermediate Filament Partnership in Skin, Skeletal Muscle, and Peripheral Nerve. Histochem. Cell Biol..

[38] Wiche G. (1998). Role of Plectin in Cytoskeleton Organization and Dynamics. J. Cell Sci..

[39] Mauldin E.A., Credille K.M., Dunstan R.W., Casal M.L. (2008). The Clinical and Morphologic Features of Nonepidermolytic Ichthyosis in the Golden Retriever. Vet. Pathol..

[40] Olivry T., Linder K.E., Wang P., Bizikova P., Bernstein J.A., Dunston S.M., Paps J.S., Casal M.L. (2012). Deficient Plakophilin-1 Expression Due to a Mutation in PKP1 Causes Ectodermal Dysplasia-Skin Fragility Syndrome in Chesapeake Bay Retriever Dogs. PLoS ONE.

[41] South A.P. (2004). Plakophilin 1: An Important Stabilizer of Desmosomes. Clin. Exp. Dermatol..

[42] Has C., Bauer J.W., Bodemer C., Bolling M.C., Bruckner-Tuderman L., Diem A., Fine J.-D., Heagerty A., Hovnanian A., Marinkovich M.P. (2020). Consensus Reclassification of Inherited Epidermolysis Bullosa and Other Disorders with Skin Fragility. Br. J. Dermatol..

[43] Bardhan A., Bruckner-Tuderman L., Chapple I.L.C., Fine J.-D., Harper N., Has C., Magin T.M., Marinkovich M.P., Marshall J.F., McGrath J.A. (2020). Epidermolysis Bullosa. Nat. Rev. Dis. Primers.

[44] Buchroithner B., Klausegger A., Ebschner U., Anton-Lamprecht I., Pohla-Gubo G., Lanschuetzer C.M., Laimer M., Hintner H., Bauer J.W. (2004). Analysis of the LAMB3 Gene in a Junctional Epidermolysis Bullosa Patient Reveals Exonic Splicing and Allele-Specific Nonsense-Mediated MRNA Decay. Lab. Investig..

[45] Liu L., Jung S.-N., Oh C., Lee K., Won H.-R., Chang J.W., Kim J.M., Koo B.S. (2019). LAMB3 Is Associated with Disease Progression and Cisplatin Cytotoxic Sensitivity in Head and Neck Squamous Cell Carcinoma. Eur. J. Surg. Oncol..

[46] Fine J.-D., Eady R.A.J., Bauer E.A., Bauer J.W., Bruckner-Tuderman L., Heagerty A., Hintner H., Hovnanian A., Jonkman M.F., Leigh I. (2008). The Classification of Inherited Epidermolysis Bullosa (EB): Report of the Third International Consensus Meeting on Diagnosis and Classification of EB. J. Am. Acad. Dermatol..

[47] Bruckner-Tuderman L., Nilssen O., Zimmermann D.R., Dours-Zimmermann M.T., Kalinke D.U., Gedde-Dahl T., Winberg J.O. (1995). Immunohistochemical and Mutation Analyses Demonstrate That Procollagen VII Is Processed to Collagen VII through Removal of the NC-2 Domain. J. Cell Biol..

[48] Dang N., Murrell D.F. (2008). Mutation Analysis and Characterization of COL7A1 Mutations in Dystrophic Epidermolysis Bullosa. Exp. Dermatol..

[49] Gache Y., Pin D., Gagnoux-Palacios L., Carozzo C., Meneguzzi G. (2011). Correction of Dog Dystrophic Epidermolysis Bullosa by Transplantation of Genetically Modified Epidermal Autografts. J. Investig. Dermatol..

[50] Baldeschi C., Gache Y., Rattenholl A., Bouillé P., Danos O., Ortonne J.-P., Bruckner-Tuderman L., Meneguzzi G. (2003). Genetic Correction of Canine Dystrophic Epidermolysis Bullosa Mediated by Retroviral Vectors. Hum. Mol. Genet..

[51] Gretzmeier C., Pin D., Kern J.S., Chen M., Woodley D.T., Bruckner-Tuderman L., de Souza M.P., Nyström A. (2022). Systemic Collagen VII Replacement Therapy for Advanced Recessive Dystrophic Epidermolysis Bullosa. J. Investig. Dermatol..

[52] Mauldin E.A. (2013). Canine Ichthyosis and Related Disorders of Cornification. Vet. Clin. N. Am. Small Anim. Pract..

[53] Guaguère É. (2008). A Practical. Guide to Canine Dermatology.

[54] Credille K.M., Barnhart K.F., Minor J.S., Dunstan R.W. (2005). Mild Recessive Epidermolytic Hyperkeratosis Associated with a Novel Keratin 10 Donor Splice-Site Mutation in a Family of Norfolk Terrier Dogs. Br. J. Dermatol..

[55] Alperin E.S., Shapiro L.J. (1997). Characterization of Point Mutations in Patients with X-Linked Ichthyosis. Effects on the Structure and Function of the Steroid Sulfatase Protein. J. Biol. Chem..

[56] Bauer A., Waluk D.P., Galichet A., Timm K., Jagannathan V., Sayar B.S., Wiener D.J., Dietschi E., Müller E.J., Roosje P. (2017). A de Novo Variant in the ASPRV1 Gene in a Dog with Ichthyosis. PLoS Genet..

[57] Bauer A., De Lucia M., Jagannathan V., Mezzalira G., Casal M.L., Welle M.M., Leeb T. (2017). A Large Deletion in the NSDHL Gene in Labrador Retrievers with a Congenital Cornification Disorder. G3 (Bethesda).

[58] Kiener S., Wiener D.J., Hopke K., Diesel A.B., Jagannathan V., Mauldin E.A., Casal M.L., Leeb T. (2021). ABHD5 Frameshift Deletion in Golden Retrievers with Ichthyosis. G3 (Bethesda).

[59] Casal M.L., Wang P., Mauldin E.A., Lin G., Henthorn P.S. (2017). A Defect in NIPAL4 Is Associated with Autosomal Recessive Congenital Ichthyosis in American Bulldogs. PLoS ONE.

[60] Guaguere E., Bensignor E., Küry S., Degorce-Rubiales F., Muller A., Herbin L., Fontaine J., André C. (2009). Clinical, Histopathological and Genetic Data of Ichthyosis in the Golden Retriever: A Prospective Study. J. Small Anim. Pract..

[61] Owczarek-Lipska M., Thomas A., André C., Hölzer S., Leeb T. (2011). Frequency of gene defects in selected European retriever populations. Schweiz. Arch. Tierheilkd..

[62] Kienesberger P.C., Oberer M., Lass A., Zechner R. (2009). Mammalian Patatin Domain Containing Proteins: A Family with Diverse Lipolytic Activities Involved in Multiple Biological Functions. J. Lipid Res..

[63] Grond S., Eichmann T.O., Dubrac S., Kolb D., Schmuth M., Fischer J., Crumrine D., Elias P.M., Haemmerle G., Zechner R. (2017). PNPLA1 Deficiency in Mice and Humans Leads to a Defect in the Synthesis of Omega-O-Acylceramides. J. Investig. Dermatol..

[64] Pichery M., Huchenq A., Sandhoff R., Severino-Freire M., Zaafouri S., Opálka L., Levade T., Soldan V., Bertrand-Michel J., Lhuillier E. (2017). PNPLA1 Defects in Patients with Autosomal Recessive Congenital Ichthyosis and KO Mice Sustain PNPLA1 Irreplaceable Function in Epidermal Omega-O-Acylceramide Synthesis and Skin Permeability Barrier. Hum. Mol. Genet..

[65] Puigdemont A., Furiani N., De Lucia M., Carrasco I., Ordeix L., Fondevila D., Ramió-Lluch L., Brazis P. (2018). Topical Polyhydroxy Acid Treatment for Autosomal Recessive Congenital Ichthyosis in the Golden Retriever: A Prospective Pilot Study. Vet. Dermatol..

[66] Nakhaei S., Heidary H., Rahimian A., Vafadar M., Rohani F., Bahoosh G.R., Amirkashani D. (2018). A New Case of Chanarin-Dorfman Syndrome with a Novel Deletion in ABHD5 Gene. Iran. Biomed. J..

[67] Eskiocak A.H., Missaglia S., Moro L., Durdu M., Tavian D. (2019). A Novel Mutation of ABHD5 Gene in a Chanarin Dorfman Patient with Unusual Dermatological Findings. Lipids Health Dis..

[68] Golda M., Mótyán J.A., Nagy K., Matúz K., Nagy T., Tőzsér J. (2020). Biochemical Characterization of Human Retroviral-Like Aspartic Protease 1 (ASPRV1). Biomolecules.

[69] Matsui T., Miyamoto K., Kubo A., Kawasaki H., Ebihara T., Hata K., Tanahashi S., Ichinose S., Imoto I., Inazawa J. (2011). SASPase Regulates Stratum Corneum Hydration through Profilaggrin-to-Filaggrin Processing. EMBO Mol. Med..

[70] Briand A., Cochet-Faivre N., Reyes-Gomez E., Jaraud-Darnault A., Tiret L., Chevallier L. (2019). NIPAL4 Deletion Identified in an American Bully with Autosomal Recessive Congenital Ichthyosis and Response to Topical Therapy. Vet. Med. Sci..

[71] Pigg M.H., Bygum A., Gånemo A., Virtanen M., Brandrup F., Zimmer A.D., Hotz A., Vahlquist A., Fischer J. (2016). Spectrum of Autosomal Recessive Congenital Ichthyosis in Scandinavia: Clinical Characteristics and Novel and Recurrent Mutations in 132 Patients. Acta Derm Venereol..

[72] Dahlqvist J., Westermark G.T., Vahlquist A., Dahl N. (2012). Ichthyin/NIPAL4 Localizes to Keratins and Desmosomes in Epidermis and Ichthyin Mutations Affect Epidermal Lipid Metabolism. Arch. Dermatol. Res..

[73] Mauldin E.A., Crumrine D., Casal M.L., Jeong S., Opálka L., Vavrova K., Uchida Y., Park K., Craiglow B., Choate K.A. (2018). Cellular and Metabolic Basis for the Ichthyotic Phenotype in NIPAL4 (Ichthyin)-Deficient Canines. Am. J. Pathol..

[74] Yen M.-C., Chou S.-K., Kan J.-Y., Kuo P.-L., Hou M.-F., Hsu Y.-L. (2018). Solute Carrier Family 27 Member 4 (SLC27A4) Enhances Cell Growth, Migration, and Invasion in Breast Cancer Cells. Int. J. Mol. Sci..

[75] Schwenk R.W., Holloway G.P., Luiken J.J.F.P., Bonen A., Glatz J.F.C. (2010). Fatty Acid Transport across the Cell Membrane: Regulation by Fatty Acid Transporters. Prostaglandins Leukot Essent Fat. Acids.

[76] Simpson J.K., Martinez-Queipo M., Onoufriadis A., Tso S., Glass E., Liu L., Higashino T., Scott W., Tierney C., Simpson M.A. (2020). Genotype-Phenotype Correlation in a Large English Cohort of Patients with Autosomal Recessive Ichthyosis. Br. J. Dermatol..

[77] Saldaña-García N., Espinosa-Fernández M.G., Serrano-Martín M.D.M., Vera Casaño Á. (2020). A new SLC27A4 mutation associated with ichthyosis prematurity syndrome and compartment syndrome. An. Pediatr..

[78] Li S., Green J.F., Jin M. (2020). Impacts of Deletion and Ichthyosis Prematurity Syndrome-Associated Mutations in Fatty Acid Transport Protein 4 on the Function of RPE65. FEBS Lett..

[79] Oji V., Traupe H. (2006). Ichthyoses: Differential Diagnosis and Molecular Genetics. Eur. J. Dermatol..

[80] Radner F.P.W., Marrakchi S., Kirchmeier P., Kim G.-J., Ribierre F., Kamoun B., Abid L., Leipoldt M., Turki H., Schempp W. (2013). Mutations in CERS3 Cause Autosomal Recessive Congenital Ichthyosis in Humans. PLoS Genet..

[81] Caroppo F., Cama E., Salmaso R., Bertolin C., Salviati L., Belloni Fortina A. (2020). A Novel KRT1 c.1433A>G p.(Glu478Gly) Mutation in a Newborn with Epidermolytic Ichthyosis. Clin. Case Rep..

[82] Nellen R.G.L., Nagtzaam I.F., Hoogeboom A.J.M., Bladergroen R.S., Jonkman M.F., Steijlen P.M., van Steensel M.A.M., van Geel M. (2015). Phenotypic Variation in Epidermolytic Ichthyosis: Clinical and Functional Evaluation of the Novel p.(Met339Lys) Mutation in the L12 Domain of KRT1. Exp. Dermatol..

[83] Al Raddadi A.A., Habibullah T.H., Abdelaal A.M., Felimban A.M., Al Raddadi H.A., Satti M.B. (2018). Epidermolytic Ichthyosis without Keratin 1 or 10 Mutations: A. Case Report. Saudi. J. Med. Med. Sci..

[84] Bannoehr J., Balmer P., Stoffel M.H., Jagannathan V., Gaschen V., Kühni K., Sayar B., Drögemüller M., Howald D., Wiener D.J. (2020). Abnormal Keratinocyte Differentiation in the Nasal Planum of Labrador Retrievers with Hereditary Nasal Parakeratosis (HNPK). PLoS ONE.

[85] Mecklenburg L., Hetzel U., Ueberschär S. (2000). Epidermolytic Ichthyosis in a Dog: Clinical, Histopathological, Immunohistochemical and Ultrastructural Findings. J. Comp. Pathol..

[86] Mauldin E.A., Elias P.M. (2021). Ichthyosis and Hereditary Cornification Disorders in Dogs. Vet. Dermatol..

[87] Peters J., Scott D.W., Erb H.N., Miller W.H. (2003). Hereditary Nasal Parakeratosis in Labrador Retrievers: 11 New Cases and a Retrospective Study on the Presence of Accumulations of Serum (‘serum Lakes’) in the Epidermis of Parakeratotic Dermatoses and Inflamed Nasal Plana of Dogs. Vet. Dermatol..

[88] Senter D.A., Scott D.W., Miller W.H., Erb H.N. (2002). Intracorneal Vacuoles in Skin Diseases with Parakeratotic Hyperkeratosis in the Dog: A Retrospective Light-Microscopy Study of 111 Cases (1973-2000). Vet. Dermatol..

[89] Weirich S., Khella M.S., Jeltsch A. (2021). Structure, Activity and Function of the Suv39h1 and Suv39h2 Protein Lysine Methyltransferases. Life.

[90] Wang L., Chakraborty D., Iqbal K., Soares M.J. (2021). SUV39H2 Controls Trophoblast Stem Cell Fate. Biochim. Biophys. Acta Gen. Subj..

[91] Dillberger J.E., Altman N.H. (1986). Focal Mucinosis in Dogs: Seven Cases and Review of Cutaneous Mucinoses of Man and Animals. Vet. Pathol..

[92] Welle M., Grimm S., Suter M., von Tscharner C. (1999). Mast Cell Density and Subtypes in the Skin of Shar Pei Dogs with Cutaneous Mucinosis. Zentralbl. Vet. A.

[93] Whipple K.M., Kieran E.A., Dark M.J., Beatty S.S. (2020). What Is Your Diagnosis? Cutaneous Mass from a Shar-Pei Dog. Vet. Clin. Pathol..

[94] Zanna G., Fondevila D., Bardagí M., Docampo M.J., Bassols A., Ferrer L. (2008). Cutaneous Mucinosis in Shar-Pei Dogs Is Due to Hyaluronic Acid Deposition and Is Associated with High Levels of Hyaluronic Acid in Serum. Vet. Dermatol..

[95] Docampo M.J., Zanna G., Fondevila D., Cabrera J., López-Iglesias C., Carvalho A., Cerrato S., Ferrer L., Bassols A. (2011). Increased HAS2-Driven Hyaluronic Acid Synthesis in Shar-Pei Dogs with Hereditary Cutaneous Hyaluronosis (Mucinosis). Vet. Dermatol..

[96] Zanna G., Docampo M.J., Fondevila D., Bardagí M., Bassols A., Ferrer L. (2009). Hereditary Cutaneous Mucinosis in Shar Pei Dogs Is Associated with Increased Hyaluronan Synthase-2 MRNA Transcription by Cultured Dermal Fibroblasts. Vet. Dermatol..

[97] Heldin P., Basu K., Kozlova I., Porsch H. (2014). HAS2 and CD44 in Breast Tumorigenesis. Adv. Cancer Res..

[98] Suzuki M., Asplund T., Yamashita H., Heldin C.H., Heldin P. (1995). Stimulation of Hyaluronan Biosynthesis by Platelet-Derived Growth Factor-BB and Transforming Growth Factor-Beta 1 Involves Activation of Protein Kinase C. Biochem. J..

[99] Vigetti D., Clerici M., Deleonibus S., Karousou E., Viola M., Moretto P., Heldin P., Hascall V.C., De Luca G., Passi A. (2011). Hyaluronan Synthesis Is Inhibited by Adenosine Monophosphate-Activated Protein Kinase through the Regulation of HAS2 Activity in Human Aortic Smooth Muscle Cells. J. Biol. Chem..

[100] Yamane T., Kobayashi-Hattori K., Oishi Y. (2011). Adiponectin Promotes Hyaluronan Synthesis along with Increases in Hyaluronan Synthase 2 Transcripts through an AMP-Activated Protein Kinase/Peroxisome Proliferator-Activated Receptor-α-Dependent Pathway in Human Dermal Fibroblasts. Biochem. Biophys. Res. Commun..

[101] Weigel P.H. (2015). Hyaluronan Synthase: The Mechanism of Initiation at the Reducing End and a Pendulum Model for Polysaccharide Translocation to the Cell Exterior. Int. J. Cell Biol..

[102] Muller and Kirk’s Small Animal Dermatology-7th Edition. https://www.elsevier.com/books/muller-and-kirks-small-animal-dermatology/miller/978-1-4160-0028-0.

[103] Rinna C., Reale G., Calafati V., Calvani F., Ungari C. (2012). Dermoid Cyst: Unusual Localization. J. Craniofac. Surg..

[104] Hong S.W. (1998). Deep Frontotemporal Dermoid Cyst Presenting as a Discharging Sinus: A Case Report and Review of Literature. Br. J. Plast. Surg..

[105] Bailey T.R., Holmberg D.L., Yager J.A. (2001). Nasal Dermoid Sinus in an American Cocker Spaniel. Can. Vet. J..

[106] van der Peijl G.J.W., Schaeffer I.G.F. (2011). Nasal Dermoid Cyst Extending through the Frontal Bone with No Sinus Tract in a Dalmatian. J. Small Anim. Pract..

[107] Burrow R.D. (2004). A Nasal Dermoid Sinus in an English Bull Terrier. J. Small Anim. Pract..

[108] Sturgeon C. (2008). Nasal Dermoid Sinus Cyst in a Shih Tzu. Vet. Rec..

[109] Bornard N., Pin D., Carozzo C. (2007). Bilateral Parieto-Occipital Dermoid Sinuses in a Rottweiler. J. Small Anim. Pract..

[110] Penrith M.L., van Schouwenburg G. (1994). Dermoid Sinus in a Boerboel Bitch. J. S. Afr. Vet. Assoc..

[111] Booth M.J. (1998). Atypical Dermoid Sinus in a Chow Chow Dog. J. S. Afr. Vet. Assoc..

[112] Cornegliani L., Jommi E., Vercelli A. (2001). Dermoid Sinus in a Golden Retriever. J. Small Anim. Pract..

[113] Camacho A.A., Laus J.L., Valéri V., Valéri F.V., Nunes N. (1995). Sinus Dermóide em Cão dos Pireneus. Braz. J. Vet. Res. Anim. Sci..

[114] Perazzi A., Berlanda M., Bucci M., Ferro S., Rasotto R., Busetto R., Iacopetti I. (2013). Multiple Dermoid Sinuses of Type Vb and IIIb on the Head of a Saint Bernard Dog. Acta Vet. Scand..

[115] Hathcock J.T., Clampett E.G., Broadstone R.V. (1979). Dermoid Sinus in a Rhodesian Ridgeback. Vet. Med. Small Anim. Clin..

[116] Lambrechts N. (1996). Dermoid Sinus in a Crossbred Rhodesian Ridgeback Dog Involving the Second Cervical Vertebra. J. S. Afr. Vet. Assoc..

[117] Antin I.P. (1970). Dermoid Sinus in a Rhodesian Ridgeback Dog. J. Am. Vet. Med. Assoc..

[118] Gammie J.S. (1986). Dermoid Sinus Removal in a Rhodesian Ridgeback Dog. Can. Vet. J..

[119] Hillbertz N.H.C.S., Andersson G. (2006). Autosomal Dominant Mutation Causing the Dorsal Ridge Predisposes for Dermoid Sinus in Rhodesian Ridgeback Dogs. J. Small Anim. Pract..

[120] Li X. (2019). The FGF Metabolic Axis. Front. Med..

[121] Luo X., Jiang Y., Chen F., Wei Z., Qiu Y., Xu H., Tian G., Gong W., Yuan Y., Feng H. (2021). ORAOV1-B Promotes OSCC Metastasis via the NF-ΚB-TNFα Loop. J. Dent. Res..

[122] Ha S.Y., Yeo S.-Y., Lee K.-W., Kim S.-H. (2021). Validation of ORAOV1 as a New Treatment Target in Hepatocellular Carcinoma. J. Cancer Res. Clin. Oncol..

[123] Jiang L., Yang H.S., Wang Z., Zhou Y., Zhou M., Zeng X., Chen Q.M. (2009). ORAOV1-A Correlates with Poor Differentiation in Oral Cancer. J. Dent. Res..

[124] Li M., Cui X., Shen Y., Dong H., Liang W., Chen Y., Hu J., Li S., Kong J., Li H. (2015). ORAOV1 Overexpression in Esophageal Squamous Cell Carcinoma and Esophageal Dysplasia: A Possible Biomarker of Progression and Poor Prognosis in Esophageal Carcinoma. Hum. Pathol..

[125] McEwan N.A., McNeil P.E., Thompson H., McCandlish I.A. (2000). Diagnostic Features, Confirmation and Disease Progression in 28 Cases of Lethal Acrodermatitis of Bull Terriers. J. Small Anim. Pract..

[126] Jiang L., Zeng X., Wang Z., Ji N., Zhou Y., Liu X., Chen Q. (2010). Oral Cancer Overexpressed 1 (ORAOV1) Regulates Cell Cycle and Apoptosis in Cervical Cancer HeLa Cells. Mol. Cancer.

[127] McEwan N.A. (2001). Malassezia and Candida Infections in Bull Terriers with Lethal Acrodermatitis. J. Small Anim. Pract..

[128] Grider A., Mouat M.F., Mauldin E.A., Casal M.L. (2007). Analysis of the Liver Soluble Proteome from Bull Terriers Affected with Inherited Lethal Acrodermatitis. Mol. Genet. Metab..

[129] Bauer A., Jagannathan V., Högler S., Richter B., McEwan N.A., Thomas A., Cadieu E., André C., Hytönen M.K., Lohi H. (2018). MKLN1 Splicing Defect in Dogs with Lethal Acrodermatitis. PLoS Genet..

[130] Binder H., Arnold S., Schelling C., Suter M., Wild P. (2000). Palmoplantar Hyperkeratosis in Irish Terriers: Evidence of Autosomal Recessive Inheritance. J. Small Anim. Pract..

[131] Plassais J., Guaguère E., Lagoutte L., Guillory A.-S., de Citres C.D., Degorce-Rubiales F., Delverdier M., Vaysse A., Quignon P., Bleuart C. (2015). A Spontaneous KRT16 Mutation in a Dog Breed: A Model for Human Focal Non-Epidermolytic Palmoplantar Keratoderma (FNEPPK). J. Investig. Dermatol..

[132] Schleifer S.G., Versteeg S.A., van Oost B., Willemse T. (2003). Familial Footpad Hyperkeratosis and Inheritance of Keratin 2, Keratin 9, and Desmoglein 1 in Two Pedigrees of Irish Terriers. Am. J. Vet. Res..

[133] Drögemüller M., Jagannathan V., Becker D., Drögemüller C., Schelling C., Plassais J., Kaerle C., Dufaure de Citres C., Thomas A., Müller E.J. (2014). A Mutation in the FAM83G Gene in Dogs with Hereditary Footpad Hyperkeratosis (HFH). PLoS Genet..

[134] Olivry T., Linder K.E. (2009). Dermatoses Affecting Desmosomes in Animals: A Mechanistic Review of Acantholytic Blistering Skin Diseases. Vet. Dermatol..

[135] Linek M., Doelle M., Leeb T., Bauer A., Leuthard F., Henkel J., Bannasch D., Jagannathan V., Welle M.M. (2020). ATP2A2 SINE Insertion in an Irish Terrier with Darier Disease and Associated Infundibular Cyst Formation. Genes.

[136] Sayyab S., Viluma A., Bergvall K., Brunberg E., Jagannathan V., Leeb T., Andersson G., Bergström T.F. (2016). Whole-Genome Sequencing of a Canine Family Trio Reveals a FAM83G Variant Associated with Hereditary Footpad Hyperkeratosis. G3 (Bethesda).

[137] Backel K.A., Kiener S., Jagannathan V., Casal M.L., Leeb T., Mauldin E.A. (2020). A DSG1 Frameshift Variant in a Rottweiler Dog with Footpad Hyperkeratosis. Genes.

[138] Rickman L., Simrak D., Stevens H.P., Hunt D.M., King I.A., Bryant S.P., Eady R.A., Leigh I.M., Arnemann J., Magee A.I. (1999). N-Terminal Deletion in a Desmosomal Cadherin Causes the Autosomal Dominant Skin Disease Striate Palmoplantar Keratoderma. Hum. Mol. Genet..

[139] Wang P., Zangerl B., Werner P., Mauldin E.A., Casal M.L. (2011). Familial Cutaneous Lupus Erythematosus (CLE) in the German Shorthaired Pointer Maps to CFA18, a Canine Orthologue to Human CLE. Immunogenetics.

[140] Mauldin E.A., Morris D.O., Brown D.C., Casal M.L. (2010). Exfoliative Cutaneous Lupus Erythematosus in German Shorthaired Pointer Dogs: Disease Development, Progression and Evaluation of Three Immunomodulatory Drugs (Ciclosporin, Hydroxychloroquine, and Adalimumab) in a Controlled Environment. Vet. Dermatol..

[141] Ferrigno A., Hoover K., Blubaugh A., Rissi D., Banovic F. (2019). Treatment of Exfoliative Cutaneous Lupus Erythematosus in a German Shorthaired Pointer Dog with Mycophenolate Mofetil. Vet. Dermatol..

[142] Hirsch D.S., Pirone D.M., Burbelo P.D. (2001). A New Family of Cdc42 Effector Proteins, CEPs, Function in Fibroblast and Epithelial Cell Shape Changes. J. Biol. Chem..

[143] Gugasyan R., Voss A., Varigos G., Thomas T., Grumont R.J., Kaur P., Grigoriadis G., Gerondakis S. (2004). The Transcription Factors C-Rel and RelA Control Epidermal Development and Homeostasis in Embryonic and Adult Skin via Distinct Mechanisms. Mol. Cell Biol..

[144] Ishida D., Su L., Tamura A., Katayama Y., Kawai Y., Wang S.-F., Taniwaki M., Hamazaki Y., Hattori M., Minato N. (2006). Rap1 Signal Controls B Cell Receptor Repertoire and Generation of Self-Reactive B1a Cells. Immunity.

[145] Chadee D.N., Kyriakis J.M. (2004). MLK3 Is Required for Mitogen Activation of B-Raf, ERK and Cell Proliferation. Nat. Cell Biol..

[146] Leeb T., Leuthard F., Jagannathan V., Kiener S., Letko A., Roosje P., Welle M.M., Gailbreath K.L., Cannon A., Linek M. (2020). A Missense Variant Affecting the C-Terminal Tail of UNC93B1 in Dogs with Exfoliative Cutaneous Lupus Erythematosus (ECLE). Genes.

[147] Correard S., Plassais J., Lagoutte L., Botherel N., Thibaud J.-L., Hédan B., Richard L., Lia A.-S., Delague V., Mège C. (2019). Canine Neuropathies: Powerful Spontaneous Models for Human Hereditary Sensory Neuropathies. Hum. Genet..

[148] Gutierrez-Quintana R., Mellersh C., Wessmann A., Ortega M., Penderis J., Sharpe S., Freeman E., Stevenson L., Burmeister L. (2021). Hereditary Sensory and Autonomic Neuropathy in a Family of Mixed Breed Dogs Associated with a Novel RETREG1 Variant. J. Vet. Intern. Med..

[149] Forman O.P., Hitti R.J., Pettitt L., Jenkins C.A., O’Brien D.P., Shelton G.D., De Risio L., Quintana R.G., Beltran E., Mellersh C. (2016). An Inversion Disrupting FAM134B Is Associated with Sensory Neuropathy in the Border Collie Dog Breed. G3 (Bethesda).

[150] Amengual-Batle P., Rusbridge C., José-López R., Golini L., Shelton G.D., Mellersh C.S., Gutierrez-Quintana R. (2018). Two Mixed Breed Dogs with Sensory Neuropathy Are Homozygous for an Inversion Disrupting FAM134B Previously Identified in Border Collies. J. Vet. Intern. Med..

[151] Jahns H., Vernau K.M., Nolan C.M., O’Neill E.J., Shiel R.E., Shelton G.D. (2020). Polyneuropathy in Young Siberian Huskies Caused by Degenerative and Inflammatory Diseases. Vet. Pathol..

[152] Plassais J., Lagoutte L., Correard S., Paradis M., Guaguère E., Hédan B., Pommier A., Botherel N., Cadiergues M.-C., Pilorge P. (2016). A Point Mutation in a LincRNA Upstream of GDNF Is Associated to a Canine Insensitivity to Pain: A Spontaneous Model for Human Sensory Neuropathies. PLoS Genet..

